# The complete chloroplast genome of *Geum macrophyllum* (Rosaceae: Colurieae)

**DOI:** 10.1080/23802359.2020.1861562

**Published:** 2021-02-03

**Authors:** Qin-Qin Li, Jun Wen

**Affiliations:** a College of Life Science and Technology, Inner Mongolia Normal University, Hohhot, PR China; b Department of Botany, National Museum of Natural History, Smithsonian Institution, Washington, DC, USA

**Keywords:** Chloroplast genome, Colurieae, *Geum macrophyllum*, Rosaceae

## Abstract

The complete chloroplast genome of *Geum macrophyllum* is reported and characterized in this study. The chloroplast genome of *G. macrophyllum* was a circular form with a size of 155,940 bp in length. The genome presented a typical quadripartite structure composed of a pair of inverted repeats (IRa and IRb) of 26,152 bp separated by a large single copy (LSC) region of 85,307 bp and a small single copy (SSC) region of 18,329 bp. The genome contained a set of 129 genes, including 84 protein-coding genes, 37 tRNA genes, and eight rRNA genes, in which 17 were duplicated and 112 were unique. Phylogenetic analysis placed *G. macrophyllum* as sister to *G. triflorum* based on current sampling.

*Geum macrophyllum* Willd. belongs to the family Rosaceae Juss., subfamily Rosoideae (Juss.) Arn., and tribe Colurieae Rydb. This species is distributed in Eurasia and North America (Rohrer 2014). According to Plants of the World online maintained by the Royal Botanic Gardens, Kew, UK (http://powo.science.kew.org), its native range is from Kamchatka to North and Central Japan, as well as North America. *G. macrophyllum* has been used as traditional medicine (McCutcheon et al. 1994; Ellsworth et al. 2013) and its roots contain compounds which are effective against fungal diseases (McCutcheon et al. 1994). The complete chloroplast (cp) genome of *G. macrophyllum* reported herein provides a foundation for further studies on its taxonomy, evolution, and population genetics.

Voucher specimen for *G. macrophyllum* (J. Wen 10,445) was collected from Quebec, Canada, and deposited in the United States National Herbarium (US). Total genomic DNA was isolated from silica gel-dried leaves using the method of Doyle and Doyle (1987). The library with insert size of 300 bp fragments was constructed using the KAPA Hyper Prep Kit for Illumina^®^ and then sequenced using the Illumina HiSeq platform in Novogene (Davis, CA). After sequencing, adapters were removed by the trimming software Trimmomatic version 0.33 (Bolger et al. 2014). The number and quality of raw paired‐end reads were evaluated by using the FastQC (Andrews 2018). The raw paired‐end reads were used to assemble the cp genome in NOVOPlasty version 3.8.2 (Dierckxsens et al. 2017), with ribulose-1, 5-bisphosphate carboxylase/oxygenase (*rbcL*) gene from *G. rupestre* (T. T. Yü et C. L. Li) Smedmark (GenBank accession no. NC_037392) as the seed sequence. Chloroplast genome annotation of *G. macrophyllum* was first performed using GeSeq (Tillich et al. 2017), with complete chloroplast genome of *G. rupestre* (NC_037392), *Fragaria chiloensis* (L.) Mill. (NC_019601) and *Farinopsis salesoviana* (Steph.) Chrtek et Soják (MT017928) as reference sequences. Draft annotation generated by GeSeq was then imported into Geneious Prime (Kearse et al. 2012) for further manual adjustment. Where necessary, gene boundaries were corrected to match the start and stop codons and intron/exon boundaries. The annotated complete cp genome of *G. macrophyllum* (accession no. MT774132) was submitted to GenBank. The complete cp genome of *G. macrophyllum* was a circular DNA molecule with a size of 155,940 bp in length. The genome had a typical quadripartite structure composed of two copies of inverted repeats (IRa and IRb: 26,152) separated by a large single-copy region (LSC: 85,307 bp) and a small single-copy region (SSC: 18,329 bp). The overall GC content was 36.6%, and that of the LSC, SSC, and each IR were 34.3%, 30.6%, and 42.6%, respectively. The cp genome encoded a set of 129 genes, including 84 protein-coding genes, 37 tRNA genes, and eight rRNA genes, in which 112 were unique and 17 were duplicated. The 17 duplicated genes in IR regions contained six protein-coding genes (*ndhB*, *rpl2*, *rpl23*, *rps7*, *rps12*, *ycf2*), seven tRNA genes (*trnA-UGC*, *trnI-CAU*, *trnI-GAU*, *trnL-CAA*, *trnN-GUU*, *trnR-ACG*, and *trnV-GAC*) and four rRNA genes (*rrn4.5*, *rrn5*, *rrn16*, and *rrn23*).

In order to investigate the phylogenetic position of *G. macrophyllum*, the cp genome sequences of 38 Rosaceae taxa were aligned with MAFFT version 7.450 (Katoh and Standley 2013) and then trimmed properly by trimAL version 1.4 (Capella-Gutiérrez et al. 2009). A maximum likelihood (ML) phylogenetic analysis was conducted using RAxML version 8 (Stamatakis 2014) following Zhang et al. (2020). The phylogenetic tree placed *G. macrophyllum* as sister to *G. triflorum* based on current sampling (Figure 1).

**Figure 1.**
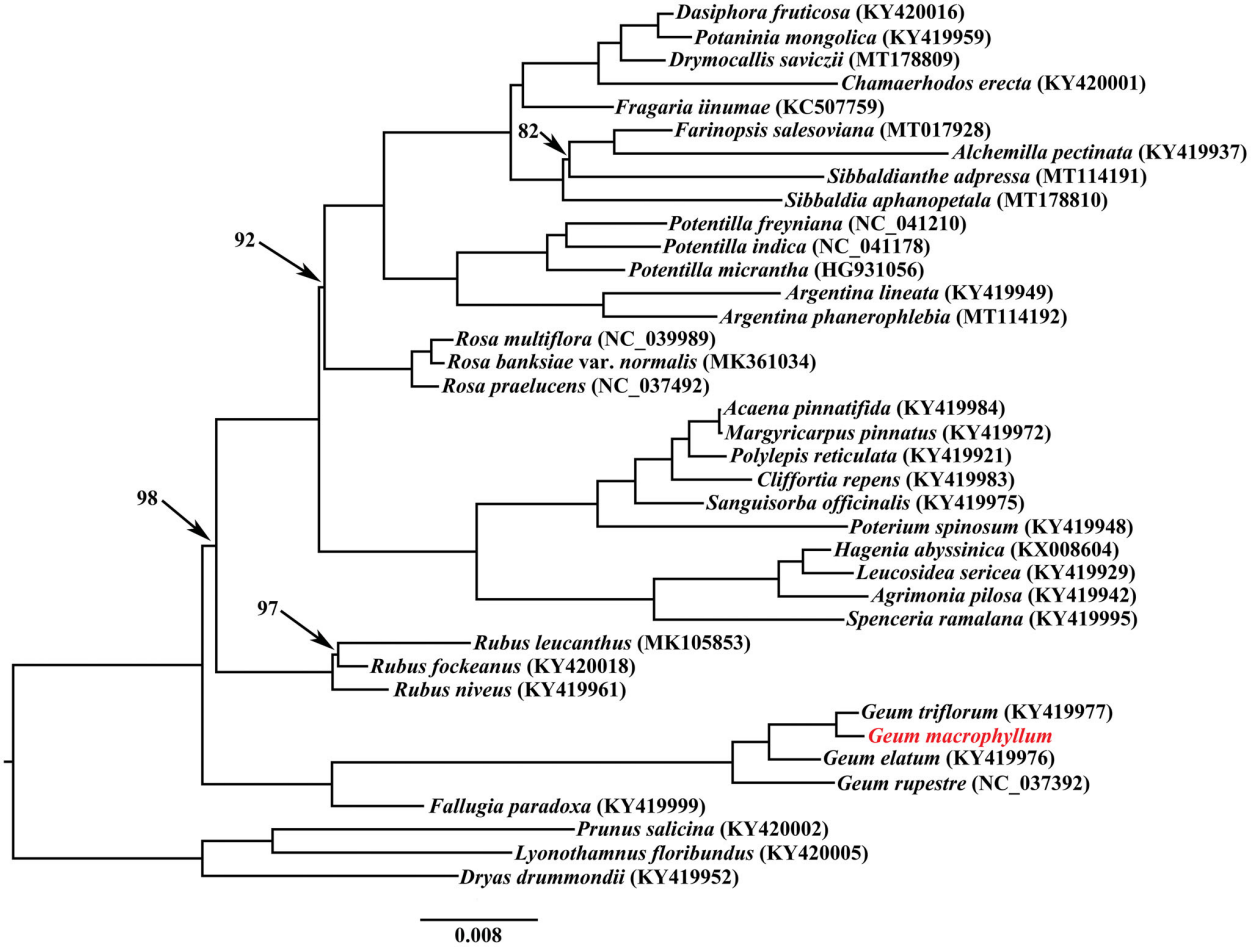
Maximum likelihood (ML) tree based on the cp genome sequences from 38 Rosaceae taxa. Branch lengths correspond to the genetic distances (substitutions per site). Values along branches correspond to ML bootstrap percentages (only values <100% are shown).

## Data Availability

The genome sequence data that support the findings of this study are openly available in GenBank of NCBI at https://www.ncbi.nlm.nih.gov/ under the accession no. MT774132. The associated BioProject, SRA, and Bio-Sample numbers are PRJNA670790, SRP288241, and SAMN16520620, respectively.
